# Supplementary material for: Identification of photoperiod-induced specific miRNAs in the adrenal glands of Sunite sheep (Ovis aries)
Source: Front Vet Sci. 2022 Jul 22;9:888207. doi: 10.3389/fvets.2022.888207 (PMC9354845; doi:10.3389/fvets.2022.888207)
Supplement: Supplementary file 1 [file Data_Sheet_1.ZIP › Supplementary file/miRDeep2 file.zip!/miRDeep2 file/Know_Structure/SPLP42A2/oar-mir-493.pdf]

miRBase precursor : oar-mir-493
Total read count : 16054
oar-miR-493-5p read count : 11464
oar-miR-493-3p read count : 4589
remaining reads : 1

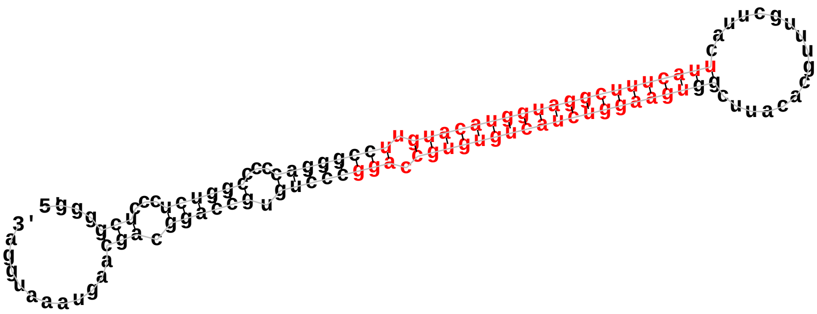

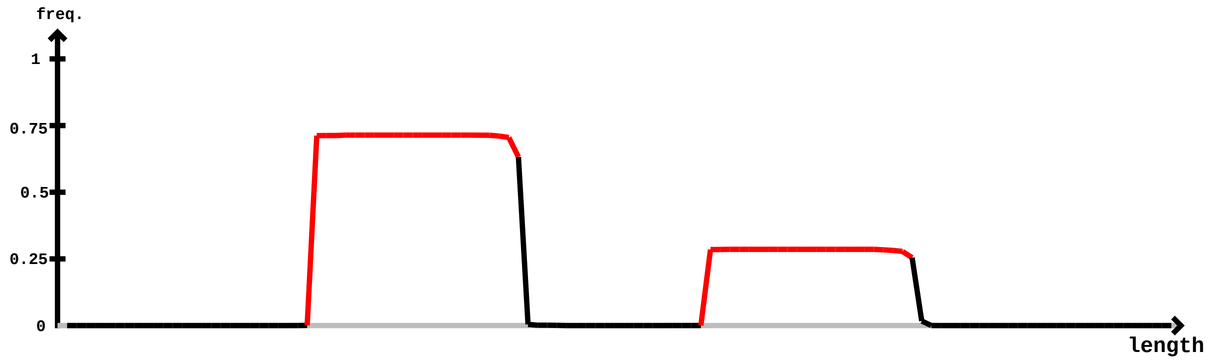


```
                              oar-miR-493-5p
                                                                      oar-miR-493-3p
5'- ggggcucccucuggcccccagggccuuguacaugguaggcuuucauucauucguuugcacauucggugaaggucuacugugugccaggcccugugccaggcagcaaguaaaugga   -3'    exp
    ...(((...((((((...((((((((.((((((((((((((((((((.................)))))))))))))))))))).)))))))).)))))).)))...........    reads     mm      sample
    .......................ccuuguacaugguaggcuuuca......................................................................    2         0       seq
    ........................cuuguacaugguaggcuuuca......................................................................    1         0       seq
    ........................cuuguacaugguaggcuuucau.....................................................................    2         0       seq
    ........................cuuguacaugguaggcuuucauu....................................................................    6         0       seq
    .........................uuguacaugguaggcuu.........................................................................    4         0       seq
    .........................uuguacaugguaggcuuu........................................................................    4         0       seq
    .........................uuguacaugguaggcuuuc.......................................................................    50        0       seq
    .........................uugCacaugguaggcuuuca......................................................................    1         1       seq
    .........................uuguacaugguaggcuuuca......................................................................    80        0       seq
    .........................uuguacaugguaggcuuucau.....................................................................    1171      0       seq
    .........................uuguacaugguaggcuuucauu....................................................................    10066     0       seq
    .........................uuguacaugguaggcuuucauuc...................................................................    47        0       seq
    ..........................uguacaugguaggcuuucau.....................................................................    1         0       seq
    ..........................uguacaugguaggcuuucauu....................................................................    5         0       seq
    ............................uacaugguaggcuuucauu....................................................................    1         0       seq
    ............................uacaugguaggcuuucauuca..................................................................    1         0       seq
    ............................uacaugguaggcuuucauucau.................................................................    9         0       seq
    ............................uacaugguaggcuuucauucauu................................................................    13        0       seq
    ..............................................................ucggugaaggucuacugugugc...............................    1         0       seq
    ..................................................................ugaaggucuacugugugc...............................    21        0       seq
    ..................................................................ugaaggucuacugugugU...............................    7         1       seq
    ..................................................................ugaGggucuacugugugc...............................    1         1       seq
    ..................................................................ugaaggucuacugugugcc..............................    36        0       seq
    ..................................................................ugaaggucuacugugugcca.............................    61        0       seq
    ..................................................................ugaaggCcuacugugugccag............................    1         1       seq
    ..................................................................ugaaggucuacugugugccag............................    374       0       seq
    ..................................................................ugaaggucuGcugugugccag............................    1         1       seq
    ..................................................................ugaagAucuacugugugccagg...........................    5         1       seq
    ..................................................................ugaaUgucuacugugugccagg...........................    1         1       seq
    ..................................................................ugaagguUuacugugugccagg...........................    5         1       seq
    ..................................................................ugaaggCcuacugugugccagg...........................    18        1       seq
    ..................................................................ugaaggucuacuguguUccagg...........................    3         1       seq
    ..................................................................ugaaggucuGcugugugccagg...........................    15        1       seq
    ..................................................................ugaaggucuacuAugugccagg...........................    2         1       seq
```

```
                         oar-miR-493-5p
                                                                  oar-miR-493-3p
ggggcucccucuggcccccagggccuuguacaugguaggcuuucauucauucguuugcacauucggugaaggucuacugugugccaggcccugugccaggcagcaaguaaaugga

..................................................................ugaaggucuacugugugccagg...........................    3753      0          seq
..................................................................ugaagguUuacugugugccaggc..........................    1         1          seq
..................................................................ugaaggucuacugugugccaggc..........................    269       0          seq
..................................................................ugaaggucuGcugugugccaggc..........................    1         1          seq
...................................................................gaaggucuacugugugccagg...........................    2         0          seq
....................................................................aaggucuacugugugccag............................    1         0          seq
....................................................................aaggucuacugugugccagg...........................    6         0          seq
....................................................................aaAgucuacugugugccaggc..........................    1         1          seq
....................................................................aaggucuacugugugccaggc..........................    3         0          seq
....................................................................aaggucuacugugugccaggcc.........................    1         0          seq
```
